# Reducing one million child deaths from birth asphyxia – a survey of health systems gaps and priorities

**DOI:** 10.1186/1478-4505-5-4

**Published:** 2007-05-16

**Authors:** Joy E Lawn, Ananta Manandhar, Rachel A Haws, Gary L Darmstadt

**Affiliations:** 1 Saving Newborn Lives Initiative, Save the Children-US, Washington, DC, USA; 2 International Perinatal Care Unit, Institute of Child Health, London, UK; 3 Health Systems Research Unit, Medical Research Council of South Africa, Cape Town, South Africa; 4 BT Research (affiliated with the Council for the Central Laboratory of the Research Councils [CCLRC] e-Science Centre, Daresbury Laboratory, UK at the time of the survey), Ipswich, UK; 5 Department of International Health, Bloomberg School of Public Health, Johns Hopkins University, Baltimore, MD, USA

## Abstract

**Background:**

Millions of child deaths and stillbirths are attributable to birth asphyxia, yet limited information is available to guide policy and practice, particularly at the community level. We surveyed selected policymakers, programme implementers and researchers to compile insights on policies, programmes, and research to reduce asphyxia-related deaths.

**Method:**

A questionnaire was developed and pretested based on an extensive literature review, then sent by email (or airmail or fax, when necessary) to 453 policymakers, programme implementers, and researchers active in child health, particularly at the community level. The survey was available in French and English and employed 5-point scales for respondents to rate effectiveness and feasibility of interventions and indicators. Open-ended questions permitted respondents to furnish additional details based on their experience. Significance testing was carried out using chi-square, F-test and Fisher's exact probability tests as appropriate.

**Results:**

173 individuals from 32 countries responded (44%). National newborn survival policies were reported to exist in 20 of 27 (74%) developing countries represented, but respondents' answers were occasionally contradictory and revealed uncertainty about policy content, which may hinder policy implementation. Respondents emphasized confusing terminology and a lack of valid measurement indicators at community level as barriers to obtaining accurate data for decision making. Regarding interventions, birth preparedness and essential newborn care were considered both effective and feasible, while resuscitation at community level was considered less feasible. Respondents emphasized health systems strengthening for both supply and demand factors as programme priorities, particularly ensuring wide availability of skilled birth attendants, promotion of birth preparedness, and promotion of essential newborn care. Research priorities included operationalising birth preparedness, effectively evaluating pregnancy risk in the community, ensuring roles for traditional birth attendants (TBAs) that link them with the health system, testing the cost-effectiveness of various community cadres for resuscitation, and developing a clear case definition for case management and population monitoring.

**Conclusion:**

Without more attention to improve care and advance birth asphyxia research, the 2 million deaths related to asphyxia, plus associated maternal deaths, will remain out of reach of effective care, either skilled or community level, for many years to come.

## Background

Birth asphyxia is the fifth largest cause of under-five child deaths (8.5%), after pneumonia, diarrhoea, neonatal infections and complications of preterm birth [1]. Birth asphyxia accounts for an estimated 0.92 million neonatal deaths annually and is associated with another 1.1 million intrapartum stillbirths [2], as well as an unknown burden of long-term neurological disability and impairment [3]. If 10 million child deaths [1] are combined with 3.2 million stillbirths [4], then birth asphyxia plus intrapartum stillbirths constitute the number-one cause of child and late fetal deaths. Yet birth asphyxia is largely invisible in health policy and programmes, and receives limited programmatic or research funding internationally [5]. Here we refer to birth asphyxia in the traditional use of the term by clinicians – the full-term baby who is not breathing and in poor condition at birth with an assumed association to acute intrapartum events. The need for more specific case definitions is apparent, but terminology discussion is not the purpose of this paper.

Recognizing that neonatal deaths (deaths in the first 28 days of life) account for almost 40% of under-five deaths, it is clear that Millennium Development Goal 4 (aiming for a two-thirds reduction of under-five mortality), cannot be met without substantially reducing neonatal deaths [6]. Neonatal and late fetal deaths are closely linked to maternal deaths, requiring common solutions. Over half of neonatal deaths occur at home in the absence of skilled care, and just three major causes account for over three-quarters of these deaths – serious infections, including tetanus (36%), complications of preterm birth (27%) and birth asphyxia (23%) [6]. Evidence exists regarding the effectiveness of interventions to reduce deaths due to neonatal infections and improve survival of small babies in the community [7-11]. However, prevention and management of birth asphyxia are much more complex at the community level and published evidence is scanty [12]. To address this gap, Saving Newborn Lives/Save the Children-US commissioned a review of birth asphyxia to include systematic global estimates, undertaken with the WHO [2]; an expert meeting [13]; and a systematic literature review for impact of interventions. Major limitations were highlighted in the scope and depth of the published literature with respect to high mortality settings with low health system coverage. We therefore undertook a targeted survey of policy makers, programme implementers and researchers, specifically selecting those involved in community-based child health programming in resource-poor settings, to identify information on current policies, programmes, practices, and health systems research priorities related to birth asphyxia.

The objectives of the survey were:

1. To identify the presence of national policies regarding newborn health;

2. To describe current methods to recognise and monitor birth asphyxia in the community;

3. To solicit opinions about the perceived appropriateness and effectiveness of interventions to address birth asphyxia, particularly at community level and specifically regarding the involvement of traditional birth attendants (TBAs) and community health workers (CHWs) in resuscitation and newborn care;

4. To discover unpublished data, lessons learned and policies relating to birth asphyxia;

5. To compile perceived gaps in programmatic implementation of proven interventions for asphyxia, especially at community level; and

6. To compile perceived knowledge gaps which limit the prevention, recognition and management of asphyxia, especially at community level.

## Methods

Survey questions were informed by a systematic literature review of the evidence base for efficacy and effectiveness of interventions to address birth asphyxia, using PubMed, POPLINE, Latin American and Caribbean Health Sciences (LILACS), BioMed Central, African Index Medicus, and WHO Regional Office for the Mediterranean (EMRO) databases. The results of this review will be published separately. The questionnaire was reviewed by an international panel of experts and adapted according to their suggestions, then pilot-tested using a convenience sample of students at the Institute of Child Health, London. A simple scale was developed to allow respondents to rate interventions and indicators for effectiveness and feasibility at community level. Effectiveness was rated from 0 (no evidence) to 5 (several randomized controlled trials). Feasibility was rated from 0 (complete infeasibility) to 5 (extreme ease of application). Additional open-ended questions encouraged respondents to provide further detail. The questionnaire was translated into French to facilitate replies from French-speaking West Africa. Total time to complete the questionnaire was approximately 1 hour.

E-mail surveys usually yield a response rate of approximately 25% or less [14]. To obtain at least 50 respondents, the questionnaire was sent to over 400 recipients active in child health care, particularly at community level in resource poor settings, including individuals from international and local NGOs addressing Safe Motherhood or newborn care; officials from relevant programmes within United Nations agencies; members of ministries of health; clinical service providers in relevant settings; academic research units publishing or currently undertaking relevant research; and individuals recommended by colleagues. To maximise the information received regarding programmes and policies, 80% of questionnaires were sent to country programme officials and policy makers. The remaining 20% were sent to researchers and global experts. The survey was disseminated by e-mail and, in some cases by airmail, followed up by two e-mail reminders. Recipients who reported difficulties accessing attachments were faxed the form (N = 8). Recipients were encouraged to forward the questionnaire to interested colleagues and/or recommend colleagues with experience addressing birth asphyxia; such individuals were added to the recipient list.

Data from returned questionnaires was entered into a Filemaker Pro version 5.5 database (Filemaker Pro Inc, Santa Clara, California, USA). Data entry was cross-checked for accuracy. Simple frequencies were calculated using Filemaker Pro; cross-tabulations were calculated using Microsoft Excel (Microsoft Office 1997, Microsoft Corp., Redmond, Washington, USA). Significance testing was carried out using chi-square, F-test and Fisher's exact probability tests as appropriate. Individuals' ratings were then compiled across the sample and mean effectiveness and feasibility scores were computed for each intervention/indicator.

## Results

The survey response rate was 44% (173/453). Respondents represented 32 countries from all 6 major regions of the world. Half (49%) of all respondents were from South Asia, and 27% and 16% were from sub-Saharan Africa and industrialised nations, respectively. All respondents from industrialised nations were affiliated with global agencies or academic institutions active in policy and/or research in developing countries or with international non-governmental organisations. The developing country respondents included ministries of health, policymakers, programme implementers, clinicians, researchers and public health/primary care workers. A high proportion (39%) reported community-based or primary healthcare experience, reflecting deliberate efforts to target these groups.

### Perceived importance of birth asphyxia

**Figure 1:**
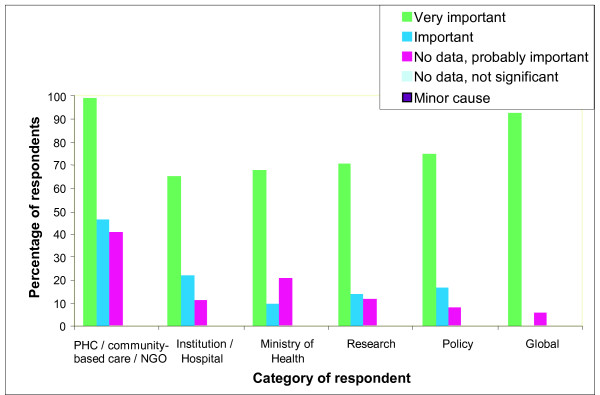
**Perceived importance of birth asphyxia by category of respondent (N = 173)**. PHC: primary health care. There was a significant difference between the perceived importance of asphyxia ("very important" or "important") for those involved in policy or research compared to those in community-level programmes (F-test = 0. 05). Those reporting uncertainty about data were significantly less likely to be involved in a programme addressing asphyxia (P = 0.009).

Almost all policy makers (93%) identified asphyxia as a major problem, whereas only 52% of community-based programme personnel had this perception (p = 0.05). An additional 21% of community-based respondents reported that birth asphyxia was "probably important," but they lacked data (Figure 1). A significantly higher proportion of individuals from industrialised countries perceived asphyxia as very important compared to those from developing countries (82% vs. 64%, respectively; p = 0.044). Respondents from sub-Saharan Africa perceived asphyxia as causing more neonatal deaths (>30%) than those from other regions (p = 0.029). Respondents who considered asphyxia "very important" were more often involved with programmes to address asphyxia than those who were unsure of the scope of the problem (p = 0.009). Among respondents not currently involved in birth asphyxia activities, 45% cited other priorities, while 40% cited financial, human resource or knowledge barriers.

### National policies

National-level newborn survival policies were reported to exist in 20 of the 27 (74%) developing countries represented, but in 8 countries, answers from different individuals were contradictory. Seventy percent of the 20 countries with policies had a perinatal and/or neonatal mortality reduction target (N = 14), and one-third (N = 7) listed asphyxia as a specific priority. Lack of data on neonatal mortality and effective interventions was the most common explanation for the lack of a national policy (37%), followed by an assumption that newborn survival was included under child survival programmes (36%), and higher priority of other issues such as malaria or HIV/AIDS (32%). A significantly higher proportion of respondents in Latin America (71% of 7) and East Asia/Pacific (75% of 4) reported a national policy in their country compared to South Asia (32% of 77), or sub-Saharan Africa (47% of 42). The few replies from North Africa and the Middle East (N = 2) precluded regional comparison.

### Programmes that address birth asphyxia

**Figure 2:**
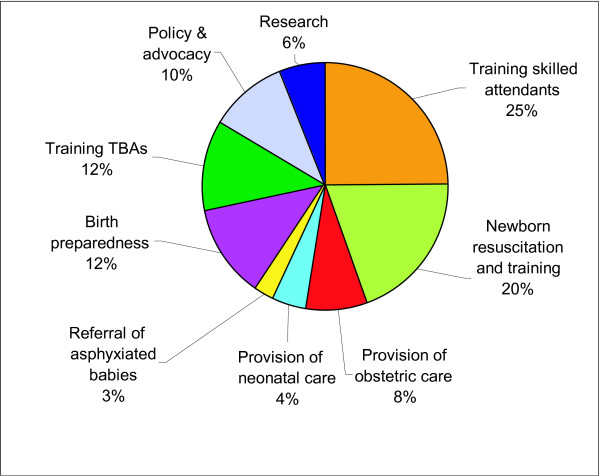
**Reported programme activities of relevance to birth asphyxia**. 142 of 173 respondents reported involvement in programmes addressing birth asphyxia, yielding 322 replies, as most programmes were implementing several relevant activities.

Reported programme experience ranged from large systems with several integrated levels of care to small, stand-alone community projects. Most respondents (88%) were actively engaged in programmes to address asphyxia. Although some interventions were newborn-specific, for example neonatal resuscitation training (N = 62, 44%), most were maternal health programmes, predominantly training of skilled birth attendants, and emergency obstetric care (Figure 2) (N = 80, 56%). South Asian programmes more often reported birth preparedness and training of skilled birth attendants or TBAs; African programmes more often reported emergency obstetric care. Community-based interventions such as birth preparedness and training of TBAs were reported by less than one-third of programmes represented, despite our pre-selected community-orientated audience. Provision of neonatal care and referral of asphyxiated babies were elements of fewer than 10% of programmes.

### Recognition of birth asphyxia in the community

**Figure 3:**
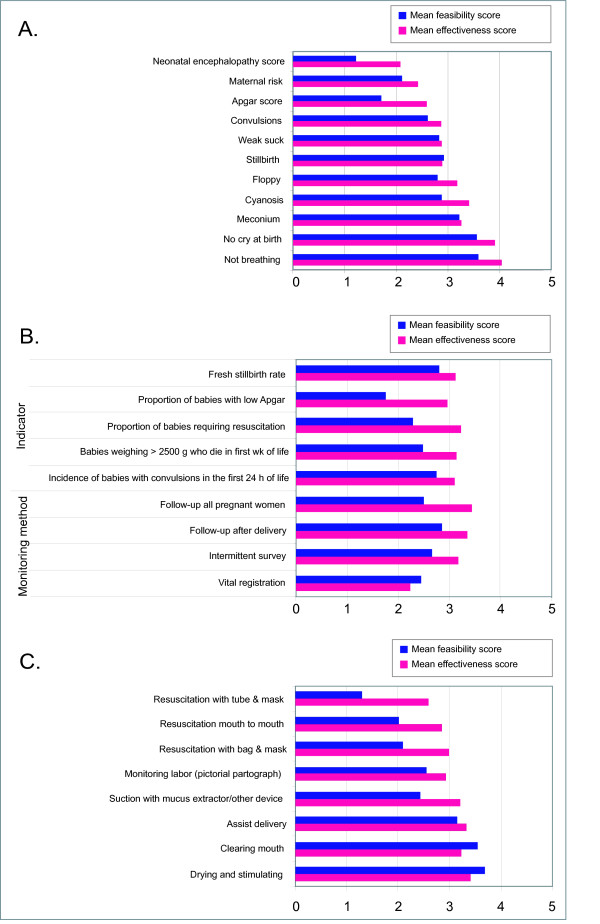
**Respondents' views on measurement and interventions for birth asphyxia**. A. Perceived effectiveness andfeasibility of signs to identify "birth asphyxia" at community level. B. Respondents' assessment of community-based asphyxia programme indicators and monitoring methods. C. Perceived effectiveness and feasibility of various birth asphyxia interventions performed by TBAs/CHWs during labour or delivery.

The most frequently reported methods for identifying the asphyxiated baby by TBAs and CHWs were "baby not crying" (55% and 61%, respectively) and "baby not breathing" (48% and 40%, respectively) at birth (Figure 3). "Not crying" and "not breathing at birth" also received the highest scores for both effectiveness and feasibility, concurring with methods already in use (Figure 2). Presence of meconium was also deemed a moderately effective and feasible sign. Other possible clinical identification methods, such as floppy baby, cyanosis, and convulsions in the first 24 hours after birth, received fairly high scores for effectiveness but lower scores for feasibility at community level (Figure 3). Apgar score, neonatal encephalopathy score, and maternal risk factor assessment received low effectiveness and feasibility scores.

### Indicators to track birth asphyxia at population level

None of the possible indicators to measure birth asphyxia at community level received high effectiveness and feasibility scores (Figure 3). Of respondents involved with programmatic activities, 35% reported that their programmes did not collect routine data on birth asphyxia. Most mentioned struggles with identifying asphyxia and collecting meaningful data to guide decisions about asphyxia interventions in the community. Among the 36% of respondents who reported that their programme collected asphyxia data, the most common method of obtaining birth asphyxia data was registration records (42%) or routine health information systems (34%). A minority used CHWs to collect data prospectively (18%), or relied on hospital records (14%), and a few (8%) conducted periodic population-based surveys. There was limited consensus about best methods for monitoring birth asphyxia incidence at the population level. While there was moderate agreement that follow-up for all pregnant women would be most effective, respondents doubted its feasibility (Fig 3b). Follow-up of all recently delivered women was considered less effective, but more feasible. Respondents considered vital registration neither effective nor feasible.

Overall, respondents considered fresh stillbirth rate the most promising indicator to track, rated as moderately effective yet feasible with training input. Onset of convulsions in the first 24 h of life and death in the first week of life in a baby weighing > 2500 g were also identified as potentially effective indicators, but difficulty obtaining complete population birthweight data limited the perceived feasibility of the latter approach (Fig 3b).

### Perceived effectiveness and feasibility of interventions during pregnancy, labour, and delivery

Essential newborn care, including stimulating, drying and warming the newborn, received the highest scores for both effectiveness and feasibility; however, impact of these interventions on asphyxia-specific deaths is unproven (Figure 3). Also highly rated were antenatal care; birth preparedness; basic obstetric care; and communicating danger signs to the family and/or TBAs/CHWs. Maternity waiting homes [15,16] and first aid for obstetric emergencies [17]were considered less effective and considerably less feasible, especially by Asian respondents. Neonatal resuscitation, maternal risk factor assessment and strengthening the referral system were all identified as highly effective, but less feasible.

### Training and roles of TBAs and CHWs

One-fourth (24%, N = 42) of respondents had experience in training TBAs and/or CHWs, and some of these provided details of the tasks TBAs and/or CHWs performed and/or the contents of their training program (N = 27). The most commonly mentioned methods for managing asphyxiated babies by community workers were drying and warming (56%), referral (46%), feeding breastmilk (42%), cleaning the mouth with gauze (13%), and ventilation (7%) using bag-and-mask, tube-and-mask or mouth-to-mouth resuscitation. Only one respondent gave results of an assessment of the effect of TBA resuscitation training on perinatal mortality [18]. Several respondents mentioned national policies that discouraged TBAs from practicing or discontinued their training:

"*...There is no more TBA training program [in] Nepal*." [Nepal]

*I train the midwives working in the primary health care centres in neonatal resuscitation practices. No evaluation has been made. According to the national health policy, home deliveries not attended by at least a midwife are not advocated and TBA's are not trained within the National Neonatal Resuscitation Training Program*." [Turkey]

When asked to rate the utility and feasibility of specific tasks being performed by TBAs and CHWs (Figure 3), most agreed that TBAs/CHWs could monitor labour, assist with delivery, and provide immediate essential newborn care, including drying, warming, and clearing the mouth with a clean cloth. The most effective and feasible postnatal interventions for the asphyxiated baby were considered to be drying and warming, and feeding breast milk (either at the breast or expressed), prior to referral. More advanced interventions, including suction with a mucus extractor or resuscitation with bag-and-mask (mouth-to-mouth if bag-and-mask was unavailable) were considered effective but less feasible. Tube-and-mask resuscitation was rated very low for feasibility, even lower than mouth-to-mouth resuscitation. Responses were mixed regarding the feasibility of TBAs performing resuscitation:

"*A simplified TBA programme, based on the WHO manuals, was used to initiate training for TBAs already in practice. Short courses and refresher courses were modified according to the experience and level of understanding of the TBAs. It gradually focused down to 'cleanliness' and ' reasons for referrals', as it took time to build up trust and understanding of basic perinatal care*." [Tanzania]

"*Most of the TBAs are illiterate and they are acceptable in the community not always because of their professional competency but [because] they belong to the community – in the training more emphasis is given to "Safe Home Delivery" rather than management at labour or care of [the] newborn (e.g., resuscitation of newborn), although basic antenatal and postnatal care [is] taught*." [India]

"*TBAs are extremely poorly trained and fairly resistant to altering practices. However, they took to bag and mask resuscitation as it was 'technology' and gave them prestige*" [Pakistan]

### Behaviour change interventions

Most respondents identified behaviour change opportunities as a major gap; 85% identified home practices as contributing to the occurrence/severity of asphyxia, while 15% identified health care system issues (Table 1). However, while 65% of the inappropriate practices in the health system were being addressed by programmes, only 27% of home practices identified as contributing to asphyxia and amenable to change were being addressed. Examples included delay in recognition of birth asphyxia by families (17%), and unsafe use of oxytocin (14%).

**Table 1:** Home and health system practices identified as contributing to occurrence or severity of birth asphyxia, and number of programmes identifying the problem who were addressing it

**Practices amenable to behaviour change**	**Number (%)**	**Behaviour change intervention implemented (% of those identifying the problem currently addressing this behaviour)**
**Home**	131 (85%)	35 (27%)
Delay in recognition by families	26 (17%)	10 (38%)
Unsafe practices at home in pregnancy and/or labour	28 (18%)	13 (46%)
Not using skilled attendant at birth/not attending ANC	10 (6%)	0 (0%)
Unsafe practices by TBA	7 (4%)	2 (29%)
Unsafe newborn care traditional practices at home	38 (25%)	0 (0%)
Incorrect use of oxytocin	22 (14%)	10 (45%)

**Health care system**	23 (15%)	15 (65%)
Unsafe practices by healthcare workers in labour	12 (8%)	7 (58%)
Unsafe newborn care practices by healthcare workers	11 (7%)	8 (73%)

Total respondents	154	50

### Priority programme and research gaps

Universally, respondents prioritised improving coverage with skilled birth attendance, followed closely by birth preparedness (Table 2). Essential newborn care and competency-based training in neonatal resuscitation were considered the next most important interventions for more widespread implementation. Eighty percent of respondents agreed on the top four priority research questions (Table 2). According to the respondents, the single most important question was the effectiveness and safety of TBAs and CHWs in newborn resuscitation.

**Table 2:** Key research and implementation priorities to address birth asphyxia, according to 173 survey respondents

*Programme *priorities	*% Naming Priority*
1. High coverage of skilled attendants at birth	(23%)
2. Promotion of birth preparedness, including emergency transport	(22%)
3. Wide availability of essential newborn care (hygiene, warmth and breastfeeding)	(19%)
4. Competency based training in neonatal resuscitation	(16%)
5. Provision of emergency obstetric care	(11%)
6. Training TBAs and CHWs where appropriate	(5%)

*Research *priorities	
1. Assess the effectiveness of TBAs/CHWs for neonatal resuscitation	(25%)
2. Evaluate the impact of birth preparedness	(23%)
3. Operations research on successful implementation/scaling-up of known interventions and roles for community cadres	(15%)
4. Accurate identification of women/neonates at risk	(15%)
5. Accurate methods for detection of "asphyxia" in the community	(7%)
6. Appropriate care of asphyxiated newborns in the community	(5%)

## Discussion

Given the lack of data regarding community-based solutions to address birth asphyxia, respondents revealed remarkable consensus on programme and research priorities. Most respondents consistently emphasized preventive Safe Motherhood strategies to reduce neonatal deaths and stillbirths due to intrapartum hypoxia (e.g., birth preparedness, presence of a skilled birth attendant, danger sign recognition) while simultaneously saving maternal lives. Most felt that newborn health programmes should initiate neonatal resuscitation activities only after establishing basic elements of essential newborn care, including drying/stimulating. Respondents also agreed that skilled birth attendants should be trained in neonatal resuscitation; however, specific roles of TBAs and/or CHWs were disputed.

### Need for better data, especially at national and sub-national level

The survey results demonstrated that birth asphyxia is perceived by those active in community level programmes as an important newborn health problem and a major public health issue, despite a dearth of data. The significant difference in perception of importance of birth asphyxia between respondents in industrialised versus developing countries, and between policy makers and community/NGO workers appears to be partially accounted for by the relatively large proportion of respondents (particularly community-based/NGO workers in developing country settings) who answered "no data but probably still important." Policy makers may have had greater access to global data on birth asphyxia, while those in-country may be unaware of or unable to access this data. This perception gap is important, particularly given the significant association found between perceived importance of asphyxia and the probability of action to address it. Those who were uninformed or unsure were significantly less likely to be involved in programmes addressing asphyxia. Local data for decision-making is clearly needed, since a large problem at global level may not be perceived as relevant locally in the absence of local data, particularly as most babies dying of asphyxia in poor communities die at home.

### Current policies and programmes

Reasons why programmes did not systematically address birth asphyxia reflect cross-cutting programmatic, financial, knowledge, and human resource constraints in settings where birth asphyxia is most common. That nearly two-thirds of the countries surveyed have a national policy for newborn health is encouraging. But given that 14% of respondents did not know if their country had a national newborn policy, many more were unsure whether the policy addressed birth asphyxia; and answers from respondents in the same country sometimes conflicted, it is clear that policies require better dissemination and ownership. The success of national policies requires dedication of key implementers, such as the targeted respondents for this survey. Confusion regarding the existence of a national policy and the specifics of its content will limit the translation of policy into programmes. More specific national goals to reduce neonatal mortality are necessary to meet Millennium Development Goal 4, and should focus attention and resources on principal causes of mortality such as birth asphyxia. More detailed national examination of policy content and wider dissemination would empower healthcare professionals, academics, NGOs and other partners to work with policy makers to support national governments in implementing these policies [19].

### Measuring birth asphyxia in communities

A major barrier to collecting quality information is the confusion and inconsistencies of asphyxia case definitions [2]. The survey results highlight this problem, as none of the possible indicators received a high score for both effectiveness (as per the evidence base) and feasibility. Fresh stillbirth rate and incidence of convulsions in the first 24 hours were considered the most favoured indicators of birth asphyxia. Despite the Western focus on neonatal encephalopathy and the fact that convulsions and/or a weak suck are among the most valid signs used in verbal autopsy algorithms to assign birth asphyxia as a cause of death [20], no TBAs and CHWs in the community used these signs to identify cases. Respondents agreed that more precise definitions of and indicators for intrapartum hypoxia and birth asphyxia are needed for programme use. Almost half the programmes implementing birth asphyxia activities made no attempt to collect birth asphyxia data. The lack of effective and feasible methods to recognise and monitor relevant outcomes for mothers and newborns in the community suggests an important need for operational programme research.

Overall, follow-up of women after delivery was considered the most effective and feasible method to collect data on asphyxia in the community, a strategy that can also increase coverage of essential interventions during the postnatal period [10], when most maternal and newborn deaths occur, while also providing an opportunity to collect outcome and coverage data. Such community-based tracking may also promote community accountability for deaths [21].

### Community-based interventions to address asphyxia

Overall, respondents favoured preventative measures and basic pregnancy and delivery care over emergency care in the community. Basic obstetric care, an improved referral system, and neonatal resuscitation all have some evidence of effectiveness [9,10,22,23], yet they received lower effectiveness and feasibility scores than essential newborn care. The challenges with these interventions, especially in isolated rural areas, are the competencies and supportive environment required [24]. Birth preparedness received high ratings, but high quality evidence of mortality impact is lacking [25]. Essential newborn care was also perceived as highly effective and feasible, but its effectiveness in reducing asphyxia deaths is unproven; benefits may be attributable primarily to skilled delivery and immediate newborn care, including drying and neonatal resuscitation.

### TBAs and CHWs: controversy and consensus

Appropriate roles of TBAs in maternal and neonatal health are hotly debated [26,27]. Although 24% of survey respondents had experience in training TBAs and/or CHWs and provided details about the training program and TBA/CHW roles, little data demonstrates the impact of TBA resuscitation training on perinatal mortality because programmes have either not collected or not reported this data. While a meta-analysis has shown a significant reduction (11%) in birth-asphyxia-attributable deaths with trained versus untrained TBAs [28], this effect was likely largely attributable to antenatal preventative measures and improved intrapartum care, as most of the TBA training schemes provided neither equipment nor specific training for neonatal resuscitation.

As intended, the survey solicited opinions from a wide-ranging audience regarding how TBAs and CHWs might address birth asphyxia. Almost all respondents felt TBAs/CHWs should be involved in newborn care, but most favoured simpler tasks. Respondents rated neonatal resuscitation by TBAs as low on effectiveness and feasibility scales. A variety of technical concerns were noted regarding proper use of resuscitation equipment by CHWs or TBAs, but virtually all respondents considered drying and stimulating the baby and clearing the mouth appropriate tasks, despite the WHO recommendation against clearing the mouth [29]. The virtually unanimous indictment of the mouth-to-tube device, which cannot be used at more than 20 breaths per minute [30], compared to the recommended 40–60 breaths per minute, suggests that use of this technology should be reviewed. Regarding care of the baby with complications of birth asphyxia, most respondents believed referral, keeping the baby dry and warm, and feeding expressed breastmilk were appropriate, emphasizing referral.

International policy changes regarding TBAs have affected the number of programmes training TBAs and it was notable in this survey that while we targeted community-based programmes, few were currently training TBAs (12%, Figure 2); however, many were training skilled attendants (25%). Ideally, all women could choose access to a skilled birth attendant, but investments of time and funds to train and sustain the vast numbers of midwives required are steep, and in some areas, infeasible. Considering these limitations, while women and babies continue to face risks in childbirth without skilled care, what interventions are possible? Can the TBAs' roles be adapted to promote birth preparedness, help women to access skilled care once they are in labour and serve as a birth companion, and provide essential immediate newborn care [22,31,32]?

### Behaviour change interventions

Ninety percent of respondents specified at least one practice amenable to behaviour change, but only about one fourth of identified behaviours (27%) were being systematically addressed. Most undesirable behaviours (85%) occurred at home, particularly unsafe traditional newborn care practices. However, efforts to address these behaviours took place predominantly within health facilities, most likely reflecting a lack of community-based workers with behaviour change training and logistical and strategic confusion about appropriate behaviour change interventions in the home. This is a major, yet potentially feasible and low-cost, implementation gap, and opportunities exist to integrate birth preparedness approaches into other existing community behaviour change messages, benefiting mothers and babies.

### Strengths and limitations of the survey

The global geographical representation of respondents is a strength of this survey. Selection to include those active in addressing birth asphyxia was deliberate, and further self-selection, with those most interested being most likely to reply, is apparent, since 88% of respondents were already involved in asphyxia-related programmes, and the response rate (44.2%) was high [14]. This does not compromise the ability of this survey to provide insight into elements of successful programmes already addressing birth asphyxia, but should be considered in interpreting the findings, since they are not representative of all maternal and child health programmes. Using e-mail for survey distribution may have limited responses, especially where e-mail access was unavailable or expensive. Translation into French clearly facilitated replies from West Africa; translation into Spanish might have increased responses from Latin America. Internal consistency checks suggested that respondents answered consistently.

While responses regarding policies and programmes were objective, the information requested on interventions and research gaps was subjective. Nevertheless, while objective evidence for effectiveness at community level is important, *perceived *effectiveness and feasibility are crucial determinants of whether existing evidence will be implemented.

## Conclusion

This survey highlights the importance of birth asphyxia as an important problem in developing country communities, accounting for more deaths than measles or malaria, yet receiving much less policy and programmatic attention. If Millennium Development Goal 4 for child survival is to be achieved, a concerted, coordinated effort is required to reduce birth asphyxia deaths by all involved along the pathway to survival, including women, families, the community, community health workers, health professionals and policy makers – this would also benefit Millennium Development Goal 5 and maternal health as well as stillbirths.

Although the survey respondents represent some of the best-developed programmes currently addressing newborn health in resource-poor settings, major gaps in current implementation are revealed. There is consensus on the need to escalate training of skilled attendants and include newborn resuscitation, and our data suggests programmes are actively doing this. However, cutting all support for TBAs risks leaving a vacuum at community level where most deliveries and most neonatal deaths still occur, areas that will wait longest to have access to skilled midwives [33]. This survey reveals strong agreement regarding more supportive and simple tasks feasible for TBAs and CHWs, as well as key home behaviours that remain unaddressed even where programmes exist. This raises a research agenda to test the ability of TBAs and CHWs to undertake less technically complex tasks than resuscitation, and to serve as links with health systems through referral and facilitating the transition to skilled care.

Respondents showed clear consensus in programme and research priorities. The international community must now act on these priorities to reduce the estimated 0.92 million neonatal deaths and 1.1 million stillbirths related to intrapartum hypoxia each year, many of which occur in the world's poorer homes [2]. With business as usual, these deaths will remain out of reach of effective care, either skilled or community level, for many years to come.

## Abbreviations

CHW, community health worker; HIV/AIDS, human immunodeficiency virus/acquired immune deficiency syndrome; NGO, non-governmental organization; TBA, traditional birth attendant; WHO, World Health Organization

## Competing interests

The author(s) declare that they have no competing interests.

## Authors' contributions

JL conceptualized the survey, co-organised the questionnaire and its distribution, designed and assisted in the analysis, co-organised the Expert Committee Meeting in Cape Town and participated in writing the paper. GD co-conceptualized the survey, co-organised the questionnaire and the Cape Town meeting, helped design the analysis framework, and participated in writing the paper. AM designed the database, analysed the data, and developed tables presented in the paper. RH participated in analysis and interpretation of data, and writing and editing the manuscript. All authors read and approved the final version of the manuscript.
